# Proposal of Early Drain Exchange After Pancreatoduodenectomy From the View of Reducing Postoperative Pancreatic Fistula

**DOI:** 10.1002/ags3.70133

**Published:** 2025-11-19

**Authors:** Taihei Soma, Mihoko Yamada, Ryo Ashida, Katsuhisa Ohgi, Shimpei Otsuka, Yoshiyasu Kato, Teiichi Sugiura, Katsuhiko Uesaka

**Affiliations:** ^1^ Division of Hepato‐Biliary‐Pancreatic Surgery Shizuoka Cancer Center Shizuoka Japan

**Keywords:** drain exchange, pancreatoduodenectomy, postoperative pancreatic fistula

## Abstract

**Background:**

To mitigate the progression of postoperative pancreatic fistula (POPF) after pancreatoduodenectomy (PD), appropriate drain management is required, and exchanging drainage tubes is commonly performed. However, the optimal timing of the first drain exchange has not yet been determined.

**Methods:**

Among 481 patients who underwent PD between 2019 and 2023, 173 (36.0%) whose drain fluid amylase activity on postoperative day (POD) 3 was ≥ 375 U/L and who underwent drain exchange were classified into 2 groups according to the timing of the first drain exchange: the early group (POD 5 or earlier, *n* = 74) and the standard group (POD 6 or later, *n* = 99). The incidence of POPF, duration of drain placement, and length of postoperative hospital stay were compared.

**Results:**

Of the overall 481 patients, grade B POPF occurred in 117 (24.3%) patients, with no grade C POPF. Among the 173 patients analyzed, the early group had a lower proportion of grade B POPF (47.3% vs. 73.7%, *p* < 0.001), shorter drain placement (21 days vs. 27 days, *p* = 0.001), and shorter hospital stay (23 days vs. 33 days, *p* = 0.003) than the standard group. A multivariate analysis revealed that performing the first drain exchange on POD 6 or later was a risk factor for grade B POPF.

**Conclusion:**

Performing the first drain exchange early after PD was associated with a reduced incidence of POPF, shorter drain duration, and shorter hospital stay. This approach may improve the drainage efficiency.

## Abstract 2

Performing the first drain exchange early after pancreatoduodenectomy was associated with a reduced incidence of postoperative pancreatic fistula, shorter drain duration, and shorter hospital stay. This approach may improve the drainage efficiency.

## 1 Introduction

Pancreatoduodenectomy (PD) is a standard procedure for the treatment of neoplasms of the duodenum, distal bile duct, and pancreatic head. Despite cumulative efforts in surgery and perioperative management, the occurrence of complications after PD remains high [1]. One of the most common complications after PD is postoperative pancreatic fistula (POPF), which has a reported incidence of 5%–26% [2, 3]. It is a potentially serious, life‐threatening event [4, 5]. For the early detection and efficient drainage of POPF, postoperative drain management is required in addition to appropriate intraoperative drain placement [6, 7]. This approach results in a shorter hospital stay, saving healthcare costs [8] and facilitates the induction of adjuvant chemotherapy without delay [9].

For patients at high risk of POPF, several efforts have been made to improve drainage efficiency, such as prophylactic continuous irrigation through the drainage tube [10, 11] and evaluation using fistulography via the drainage tube [12, 13]. In addition, exchanging the drainage tube is considered effective in adjusting the drain position and size, as well as in preventing drain occlusion [13, 14]. However, no previous reports have specifically focused on the timing of drain exchange. This decision involves a clinical dilemma: exchanging in the early postoperative period may be associated with a risk of drain deviation with insufficient fistula formation, whereas exchanging in the late period could result in drain occlusion and retrograde infection.

We conducted this retrospective study based on the hypothesis that prolonged placement of the same drainage tube increases the risk of drain occlusion and retrograde infection, resulting in delayed recovery from POPF. This study aimed to review the postoperative clinical course according to the timing of the first drain exchange and to clarify the optimal timing of the first drain exchange after PD.

## 2 Materials and Methods

### 2.1 Patients

**FIGURE 1:**
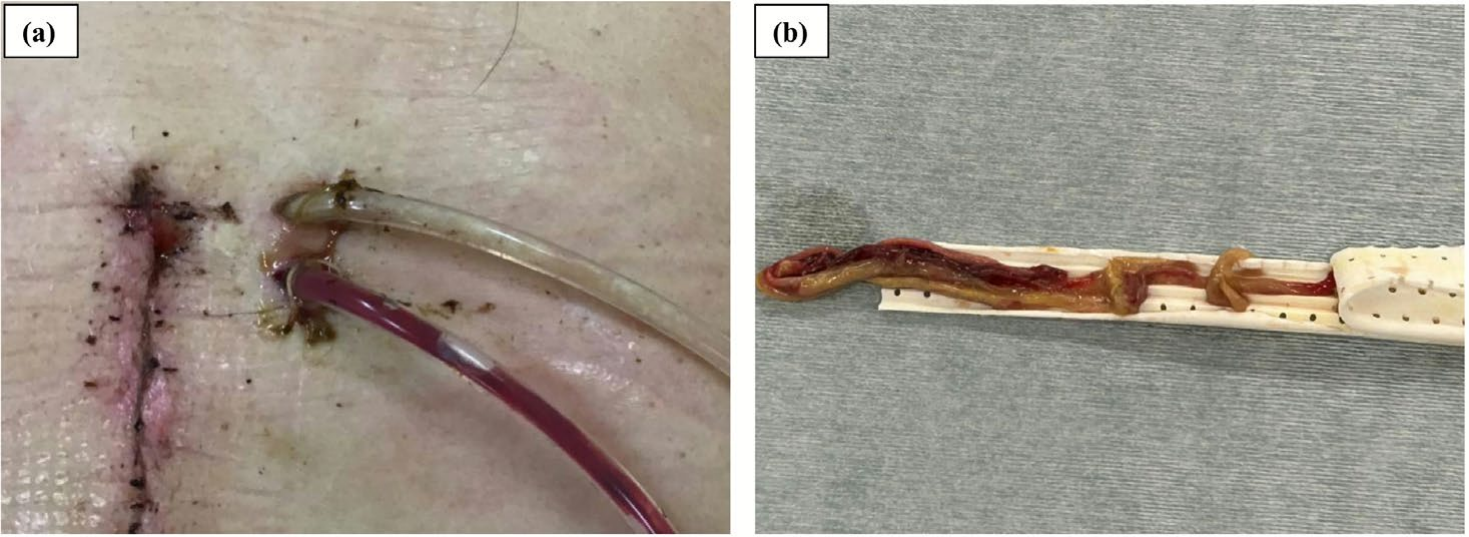
Example photographs of drain occlusion. (a) The upper drain is occluded by viscous pus with infection. (b) An accumulated fibrin clot was discharged during drain exchange.

**FIGURE 2:**
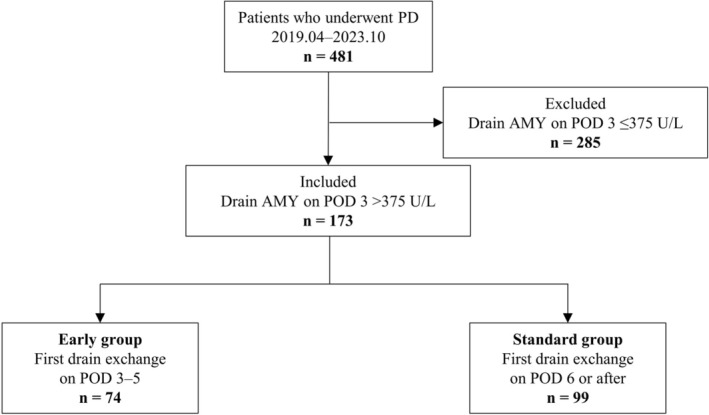
Consort flowchart of this study. AMY, amylase; BL, biochemical leakage; DM, diabetes mellitus; PD, pancreatoduodenectomy; POD, postoperative day; POPF, postoperative pancreatic fistula.

Between April 2019 and October 2023, 481 patients underwent PD at our hospital. Among them, we retrospectively analyzed 173 patients (36.0%) whose amylase activity in the drain fluid on POD 3 exceeded 3 times the normal serum range (375 U/L) at sites anterior or posterior to the pancreaticojejunal anastomosis site, corresponding to a biochemical leak (BL) as defined by the International Study Group on Pancreatic Surgery (ISGPS) criteria [15]. All patients underwent drain exchange. At our institution, drain exchange had been traditionally performed on POD 7, with some variety (e.g., POD 6 or 8), but this was changed to POD 4 (or alternatively POD 3 or 5) in July 2021 with the aim of reducing the risk of drain occlusion due to fibrin clots (Figure 1 shows an example of a drain occlusion). To clarify the association between the timing of the first drain exchange and the incidence of POPF, patients were classified into 2 groups: 74 patients who underwent the first drain exchange on POD 5 or earlier (early group) and 99 patients who underwent the first drain exchange on POD 6 or later (standard group) (Figure 2).

This study was approved by the Institutional Review Board of Shizuoka Cancer Center (approval number J2022‐189‐2022‐1‐3).

### 2.2 Surgical Procedure

**FIGURE 3:**
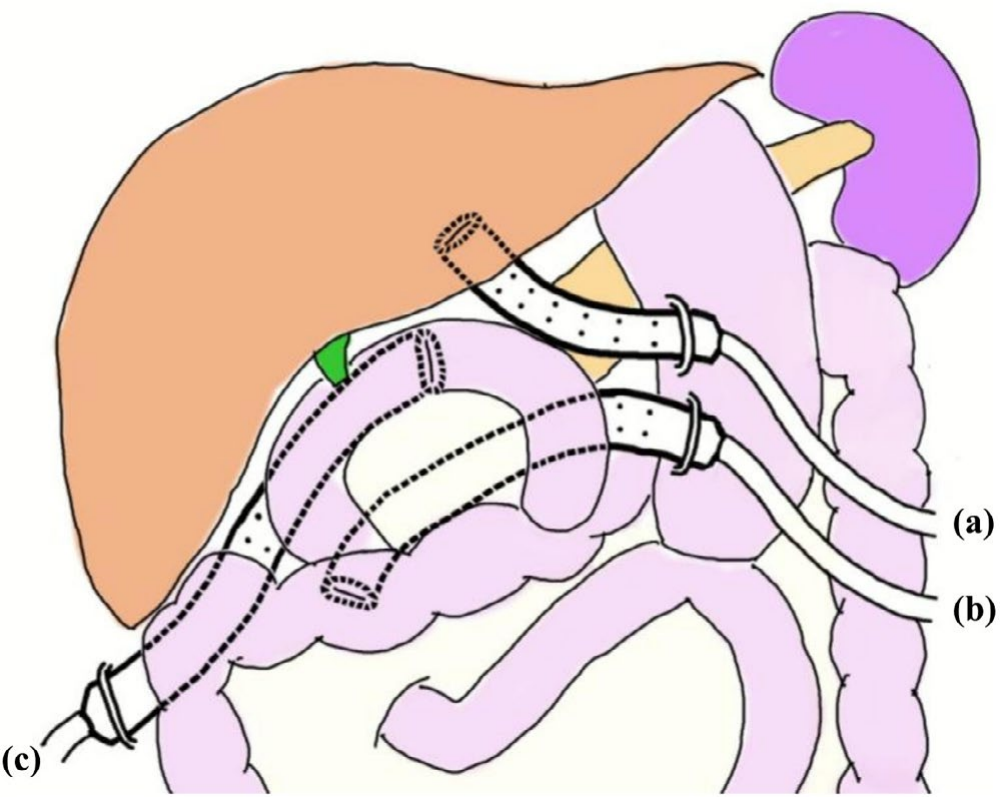
Schematic illustration of intraoperative drain placement in pancreatoduodenectomy. (a) Anterior to pancreaticojejunal anastomoses. (b) Posterior to pancreaticojejunal anastomoses. (c) Posterior to choledochojejunal anastomosis.

Our standard procedure was PD or subtotal stomach‐preserving PD and reconstruction using the modified Child's method. Pancreatojejunostomy was performed using the modified Blumgart method [16]. In principle, pancreaticojejunal anastomoses were stented externally from April 2019 to December 2022 and converted to internal stents in January 2023. As prophylactic drainage tubes, closed and continuous suction drainage systems (Davol Reliavac Silicone Drain, C. R. Bard Inc., Murray Hill, NJ, USA) were routinely placed anterior and posterior to the sites of pancreaticojejunal anastomoses, and posterior to the site of choledochojejunal anastomosis (Figure 3) [17]. Ceftriaxone or cefazoline was administered as perioperative antimicrobial prophylaxis until POD 1 [18]. In cases where preoperative bile cultures were obtained, the antimicrobial prophylaxis regimen was modified based on bacterial findings. At the end of the operation, peritoneal lavage was performed using 7000 mL of normal saline, and the fluid from the final lavage was collected for bacterial culturing [19].

### 2.3 Postoperative Drain Management

Amylase activity in the fluid from the drains anterior and posterior to the pancreaticojejunal anastomosis was measured on POD 1 and 3. If amylase activity on POD 3 was less than or equal to three times the upper institutional normal serum amylase level, the drain fluid was visibly clear, and there was no other evidence of complications, all drains were removed on POD 4. In patients whose drains were left in place, drains both anterior and posterior to the pancreaticojejunal anastomosis were exchanged over a guide wire and managed as passive gravity drainage. Amylase activity in drain fluid was measured on the day following the first exchange, although this was not feasible in all cases. After the first exchange, fistulography and drain exchange were performed once or twice a week. When the drain output decreased, the drain became open. The drains were removed after the attending surgeon determined that the POPF had completely healed, which was defined as minimal and non‐purulent drain output, no evidence of inadequate drainage on imaging, and no fever or elevated inflammatory markers. Blood tests were scheduled on POD 1, 3, 6, and 10; however, additional tests could be performed or omitted depending on the postoperative course. Postoperative CT scan was generally performed in patients clinically suspected to have insufficient drainage or uncontrolled infection. Patients were discharged when they were able to consume more than half of their meals, showed no signs of elevated inflammatory markers, and regained an activity level sufficient for independent living at home. In principle, all drains should be removed before discharge. However, if drainage is adequate, the output is low, and the patient is capable of managing the drain independently, discharge with the drain in place may be permitted.

### 2.4 Definition of Postoperative Complications

POPF was diagnosed based on the revised International Study Group on Pancreatic Surgery (ISGPS) definition [15]. BL was diagnosed if amylase activity in the drain fluid was more than 3 times the upper limit of the institutional normal serum amylase activity (375 U/L) on POD 3. BL may progress to POPF in patients with clinically relevant conditions. The diagnosis of grade B POPF was based on a change in postoperative management, such as additional percutaneous interventions and arterial embolization for bleeding, or persistent drain placement for > 21 days. The diagnosis of grade C POPF was based on the development of organ failure, the necessity for reoperation, or death. If drains were removed within 21 days after the operation, patients were diagnosed with BL, even if multiple simple drain exchanges were required.

### 2.5 Risk Classification of POPF

Each patient was assessed for the risk of POPF using the clinical risk score by Callery et al., a 10‐point scale consisting of main pancreatic duct (MPD) diameter, pancreatic texture, pathological findings, and intraoperative blood loss [3], as well as risk classification by ISGPS, which stratifies patients into four groups according to MPD diameter and pancreatic texture [20].

### 2.6 Statistical Analyses

Differences in the numerical data were examined using the *χ* ^2^ test or Fisher's exact test, where appropriate. Quantitative variables were expressed as medians and evaluated using the Mann–Whitney *U* test for analyses between the two groups. To identify the risk factors for grade B POPF, previously known risk factors [3, 21, 22, 23], along with the timing of the first drain exchange, were included in a univariate analysis, and those with statistical significance were subsequently entered into a multivariate logistic regression analysis. Statistical significance was set at *p* < 0.05. All statistical analyses were performed using EZR (Saitama Medical Center, Jichi Medical University, Saitama, Japan) [24].

## 3 Results

### 3.1 Baseline Characteristics, Operative Details, and Risk Classification of POPF

The baseline characteristics and operative details are shown in Table 1. Presence of diabetes mellitus, type of MPD stent on pancreaticojejunal anastomosis, operative time, and positive rate in intraoperative bile culture were significantly different between the two groups. In the risk classification of POPF, the clinical scores by Callery et al. (*p* = 0.565) and classification by ISGPS (*p* = 0.699) were comparable between the two groups.

**TABLE 1:** Baseline characteristics and operative details in study cohort.

	Early group *n* = 74	Standard group *n* = 99	*p*
Baseline character			
Age (year)^a^	71 (45–86)	71 (30–84)	0.924
Sex, male	55 (74.3%)	60 (60.6%)	0.073
BMI (kg/m^2^)^a^	23.0 (14.1–29.2)	23.1 (17.4–35.5)	0.469
Disease			
Pancreatic cancer	29 (39.2%)	20 (20.2%)	0.066
Biliary duct cancer	28 (37.8%)	40 (40.4%)	
Other	17 (23.0%)	39 (39.4%)	
Diabetes mellitus	**21 (28.4%)**	**12 (12.1%)**	**0.010**
Neoadjuvant chemotherapy	16 (21.6%)	13 (13.0%)	0.152
Preoperative biliary drainage	40 (54.1%)	48 (48.5%)	0.539
Albumin (g/dL)^a^	4.0 (2.6–4.9)	4.1 (2.4–5.2)	0.220
MPD diameter (mm)^a^, ^b^	3 (1–9)	3 (0.50–10)	0.989
Operative details			
PV/SMV resection	8 (10.8%)	13 (13.1%)	0.815
Pancreatic texture, soft	61 (82.4%)	85 (85.9%)	0.673
MPD stent, internal	**37 (50.7%)**	**7 (7.1%)**	**< 0.001**
Operative time (min)^a^	**459 (285–697)**	**431 (263–794)**	**0.019**
Blood loss (mL)^a^	638 (152–3927)	597 (185–4447)	0.628
Intraoperative culture			
Bile, positive	**51 (68.9%)**	**53 (53.5%)**	**0.043**
Peritoneal lavage, positive	11 (14.9%)	9 (9.2%)	0.337
Risk assessment for POPF			
Clinical Score by Callery et al.^a^	6 (1–10)	6 (0–10)	0.565
Classification by ISGPS			0.699
A	7 (9.5%)	10 (10.1%)	
B	6 (8.1%)	4 (4.0%)	
C	14 (18.9%)	22 (22.2%)	
D	47 (63.5%)	63 (63.6%)	

*Note:* Bold values denote statistical significance at the *p* < 0.05 level.

Abbreviations: BMI, body mass index; CT, computed tomography; ISGPS, International Study Group on Pancreatic Surgery; MPD, main pancreatic duct; PD, pancreatoduodenectomy; POD, postoperative day; POPF, postoperative pancreatic fistula; PV, portal vein; SMV, superior mesenteric vein.

^a^ Median (range).

^b^ Measured on preoperative CT scan.

### 3.2 Postoperative Drain Fluid Examinations and Outcomes

Among the 481 patients who underwent PD during the study period, 117 (24.3%) had grade B POPF. Furthermore, grade B POPF was observed in 80 of 234 patients (34.2%) who underwent PD before June 2021 and in 37 of 247 patients (15.0%) who underwent PD on or after June 2021. None of the patients had grade C POPF.

Table 2 shows details of postoperative drain fluid tests and postoperative outcomes among analyzed 173 patients whose amylase activity in drain fluid on POD 3 exceeded 375 U/L and who underwent drain exchange. The amylase activity in the drain fluid on POD 1 and 3 was comparable between the two groups. In the early group, the incidence of grade B POPF was lower in comparison to the standard group (47.3% vs. 73.7%, *p* < 0.001). In both groups, no patients experienced drain deviation or bleeding during drain exchange. Related to the standard group, the duration of drain placement (21 days vs. 28 days, *p* = 0.001) and postoperative hospital stay (26 days vs. 33 days, *p* = 0.003) were shorter in the early group.

**TABLE 2:** Postoperative drain fluid test and postoperative outcomes.

	Early group *n* = 74	Standard group *n* = 99	*p*
Postoperative drain fluid test			
Amylase activities (U/L)			
POD 1^a^	8399 (795–87 192)	8267 (1047–67 771)	0.356
POD 3^a^	1742 (403–16 429)	1887 (398–25 943)	0.349
Drain fluid culture, positive			
POD 1	19 (25.7%)	21 (21.2%)	0.585
POD 3	30 (40.5%)	28 (28.3%)	0.105
Postoperative outcomes			
POPF			
Grade B	**35 (47.3%)**	**73 (73.7%)**	**< 0.001**
Grade C	0 (0.0%)	0 (0.0%)	
Reoperation	0 (0.0%)	1 (1.0%)	1.000
Drain exchange frequency^a^	3 (1–13)	4 (1–14)	0.858
Drain deviation during exchange	0 (0.0%)	0 (0.0%)	1.000
Bleeding during exchange	0 (0.0%)	0 (0.0%)	1.000
Drain re‐insertion	4 (5.4%)	3 (3.0%)	0.463
On the day of drain removal (POD)^a^	**21 (6–104)**	**28 (8–97)**	**0.001**
Postoperative in‐hospital stay (POD)^a^	**26 (9–61)**	**33 (14–71)**	**0.003**
Discharge with drain placement	5 (6.8%)	5 (5.1%)	0.746

*Note:* Bold values denote statistical significance at the *p* < 0.05 level.

Abbreviations: POD, postoperative day; POPF, postoperative pancreatic fistula.

^a^ Median (range).

### 3.3 Univariate and Multivariate Analysis for Grade B POPF

Table 3 shows the results of univariate and multivariate analyses for Grade B POPF. In the multivariate analysis, high BMI (odds ratio [OR], 5.14; 95% confidence interval [CI], 1.75–15.10; *p* = 0.015), positive drain culture on POD 1 (OR, 4.67; 95% CI, 1.54–14.2; *p* = 0.007), and first drain exchange on POD 6 or later (OR, 4.06; 95% CI, 1.77–9.29; *p* < 0.001) were each identified as significant risk factors for Grade B POPF.

**TABLE 3:** Univariate and multivariate analyses of risk factors for Grade B POPF in patients whose amylase activity in fluid from drain on POD 3 after PD ≥ 375 U/L and who underwent drain exchange.

Variables	Grade B POPF					
	(−)	(+)	Univariate	Multivariate		
	*n* = 65	*n* = 108	*p*	OR	95% CI	*p*
BMI (> 25 kg/m^2^)	**5 (10.0%)**	**32 (34.4%)**	**0.001**	**5.14**	**1.75–15.10**	**0.015**
Diabetes mellitus (presence)	10 (15.4%)	23 (21.3%)	0.425			
Disease (not pancreatic cancer)	46 (70.8%)	78 (72.2%)	0.863			
MPD diameter (< 3 mm)	25 (38.5%)	43 (39.8%)	0.874			
Pancreatic texture (soft)	54 (83.1%)	92 (85.2%)	0.829			
Type of MPD stent (external)	44 (67.7%)	84 (77.8%)	0.156			
Blood loss (> 1000 mL)	8 (12.3%)	24 (22.2%)	0.111			
Drain amylase on POD 1 (> 10 000 U/L)	22 (33.8%)	51 (47.2%)	0.112			
Drain culture on POD 1 (positive)	**8 (12.3%)**	**32 (29.6%)**	**0.009**	**4.67**	**1.54–14.2**	**0.007**
Timing of the first drain exchange (POD 6 or later)	**26 (40.0%)**	**73 (67.6%)**	**< 0.001**	**4.06**	**1.77–9.29**	**< 0.001**

*Note:* Bold values denote statistical significance at the *p* < 0.05 level.

Abbreviations: BMI, body mass index; CI, confidence interval; MPD, main pancreatic duct; OR, odds ratio; PD, pancreatoduodenectomy; POD, postoperative day; POPF, postoperative pancreatic fistula.

### 3.4 Amylase Activity in the Drain Fluid, Drain Fluid Output Volume, and Serum Inflammatory Markers

Amylase activity in the drain fluid was compared in patients for whom measurements were obtained both before and after the first drain exchange: 73 of 74 patients (98.6%) in the early group and 55 of 99 patients (55.6%) in the standard group.

**FIGURE 4:**
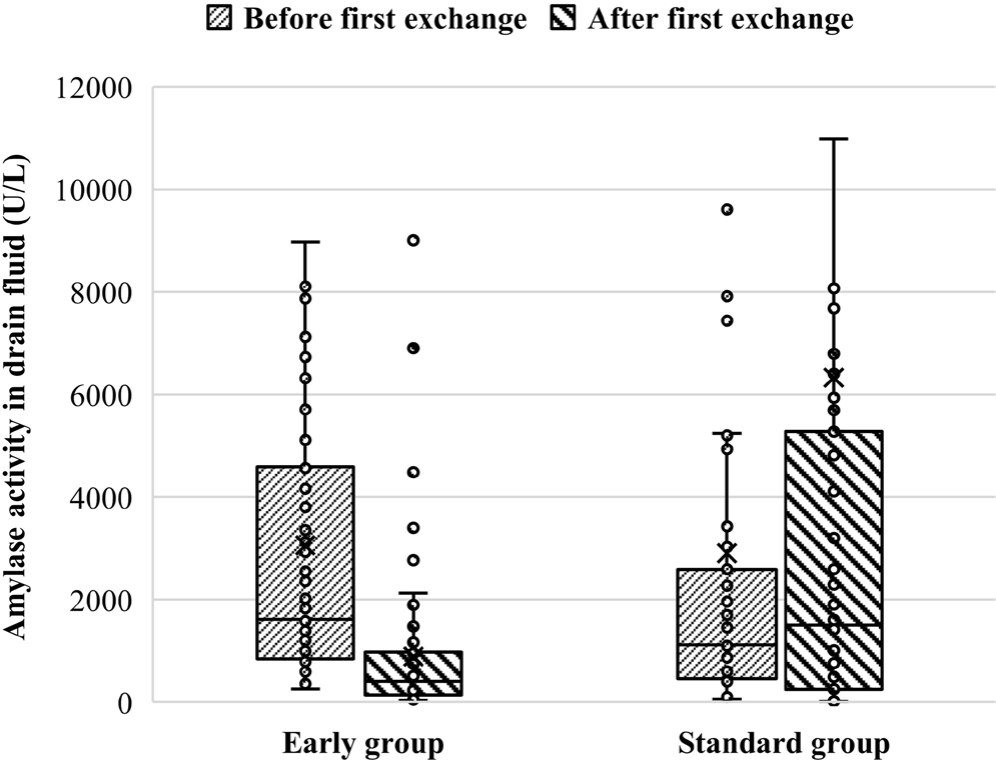
Box plot of changes in amylase activity in drain fluid from before to after the first drain exchange.

Figure 4 shows a box plot of the amylase activity in the drain fluid in each group. From before to after the first drain exchange, amylase activity decreased in the early group (median, from 1610 to 404 U/L), whereas it tended to increase in the standard group (median, from 1112 to 1500 U/L). The median change in amylase activity before to after the first drain exchange was −1119 U/L in the early group and + 438 U/L in the standard group, respectively (*p* < 0.001). As an additional analysis, the median change in drain fluid output volume before and after the first drain exchange was ±0 mL in the early group and −18 mL in the standard group (*p* = 0.061).

Figure S1 shows the postoperative trends in serum CRP and WBC counts. There were no remarkable differences in CRP levels or WBC counts between the two groups.

## 4 Discussion

This is the first report on the optimal timing of the first drain exchange in patients to reduce POPF after PD. In patients whose amylase activity in the drain fluid on POD 3 exceeded three times the normal serum range after PD, performing the first drain exchange in the early period (POD 5 or earlier) was associated with less grade B POPF, a shorter duration of drain placement, and shorter postoperative hospital stay relative to the standard period (POD 6 or later). This approach may improve drainage efficiency. Following the first drain exchange, amylase activity in drain fluid decreased in the early group, whereas it increased in the standard group. This difference of trends may suggest that most patients in the early group underwent drain exchange before occlusion, whereas some patients in the standard group had drains left in place after occlusion due to prolonged placement, resulting in the accumulated pancreatic fluid being drained after the exchange. When examining all patients who underwent PD during the study period, the incidence of grade B POPF improved from 34.2% to 15.0% after the introduction of early first drain exchange in July 2021. This outcome compares favorably with previously reported results [2, 20]. The reduction in POPF and shorter hospitalization associated with early drain exchange may contribute to timely initiation and an increased completion rate of adjuvant chemotherapy [25].

In previous studies related to drain management after PD, it was commonly accepted that in patients at low risk for POPF, drains should be removed in the early postoperative period to prevent retrograde intra‐abdominal infection and shorten hospital stay [7, 26, 27]. However, in patients at a high risk for POPF, there is no definitive consensus on drain management. Although some studies suggest that drain irrigation reduces POPF, a randomized trial showed no benefit [10, 11, 28]. The effectiveness of closed suction versus passive drainage remains unclear [29, 30, 31]. Fistulography and daily flushing may aid in the POPF assessment and drain patency [13, 32]. Our approach of conducting the first drain exchange in the early period allows for the visual confirmation of obstructions and their immediate resolution, thereby improving drainage efficiency. This proactive management strategy may be beneficial for preventing the progression of biochemical leak to grade B POPF in patients undergoing PD. Although early drain exchange was hypothesized to prevent obstruction, drain fluid output decreased more in the Standard group, possibly due to differences in postoperative timing or reduced ascites after obstruction relief. No significant differences were observed in CRP or WBC, likely because surgical factors and complications such as POPF had a greater impact on these nonspecific markers.

Concerns associated with the first drain exchange in the early period after PD were drain deviation due to inadequate fistula formation and bleeding during exchange. In our institution, intraoperative drains are placed in a straight line in anticipation of a subsequent exchange. To avoid bleeding, relatively soft silicone flat drains were placed without contact with the portal vein or artery. Even in cases with an insufficient fistula in the early postoperative period, careful use of a relatively stiff guidewire can be performed without any deviation. No troublesome bleeding during the drain exchange was observed during the study period.

The present study was associated with several limitations, in addition to its retrospective design and single center setting. First, routine imaging studies were not performed before the first drain exchange. Thus, unnecessary drain exchanges might have been performed. Some of the patients in the early group might have originally undergone drain removal on POD 5–6 in patients in the standard group. Nevertheless, we believe that this conservative policy helped us to avoid lethal grade C POPF. Second, as this study mainly compared different study periods, there were some differences between the two groups in terms of patient background, surgical techniques, and perioperative management. In particular, the difference in the type of MPD stent is a major limitation. To adjust for these differences, both univariate and multivariate analysis were performed, which identified an association between the timing of the first drain exchange and the incidence of grade B POPF. Third, while we hypothesized that early drain exchange would reduce the risk of drain occlusion and retrograde infection, we did not directly analyze these outcomes. Before the first drain exchange, CT scans were performed in 21.6% of patients in the Early group and 37.4% in the Standard group (*p* = 0.031), but these selective data are biased. Further investigations are required to confirm this effect.

In conclusion, the first drain exchange in the early period after PD was associated with a reduced incidence of grade B POPF, leading to a shorter drain duration and hospital stay. This approach may improve the drainage efficiency and help mitigate the progression of POPF.

## Author Contributions

**Taihei Soma:** conceptualization, methodology, data curation, investigation, validation, formal analysis, project administration, writing – original draft, resources. **Mihoko Yamada:** conceptualization, methodology, data curation, resources, formal analysis, project administration, validation, supervision, writing – original draft. **Ryo Ashida:** writing – review and editing. **Katsuhisa Ohgi:** writing – review and editing. **Shimpei Otsuka:** writing – review and editing. **Yoshiyasu Kato:** writing – review and editing. **Teiichi Sugiura:** writing – review and editing. **Katsuhiko Uesaka:** writing – review and editing.

## Ethics Statement

The study was approved by the Institutional Research Ethics Committee (J2022‐189‐2022‐1‐3) and with the 1964 Helsinki Declaration and its later amendments or comparable ethical standards.

## Consent

The authors have nothing to report.

## Conflicts of Interest

The authors declare no conflicts of interest.

## Supporting information


**Figure S1:** Postoperative trends in serum CRP (a) and WBC count (b). CRP, C‐reactive protein; POD, postoperative day; WBC, white blood cell.
