# Supplementary figures and images for: Proposal of Early Drain Exchange After Pancreatoduodenectomy From the View of Reducing Postoperative Pancreatic Fistula
Source: Ann Gastroenterol Surg. 2025 Nov 19;10(3):811–8. doi: 10.1002/ags3.70133 (PMC13178272; doi:10.1002/ags3.70133)

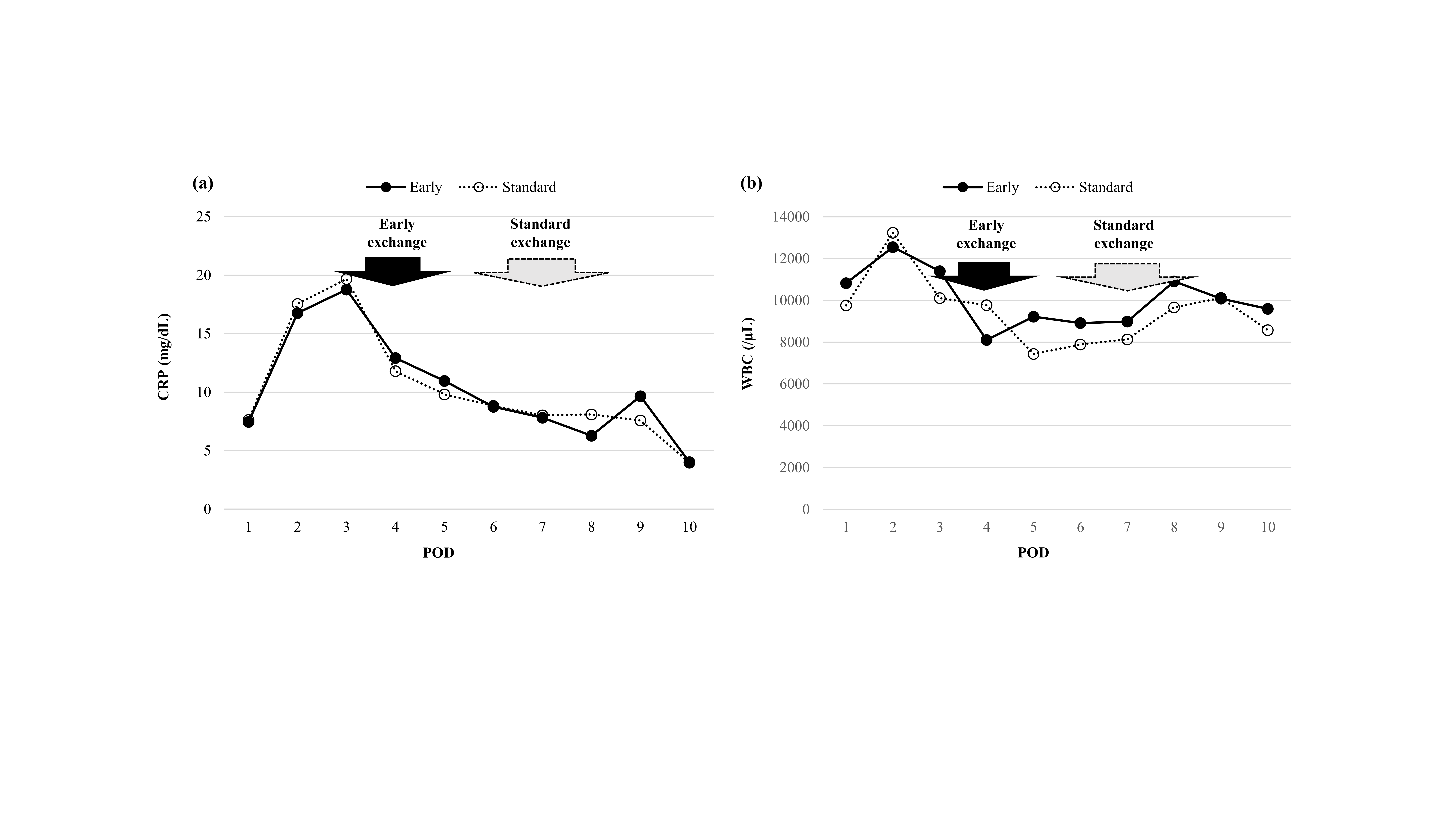

Supplement: Supplementary file 1 — Figure S1: Postoperative trends in serum CRP (a) and WBC count (b). CRP, C‐reactive protein; POD, postoperative day; WBC, white blood cell. [file AGS3-10-811-s001.tif]
